# Comprehensive Analysis Revealed that *CDKN2A* is a Biomarker for Immune Infiltrates in Multiple Cancers

**DOI:** 10.3389/fcell.2021.808208

**Published:** 2021-12-23

**Authors:** Zheng Chen, Yingjie Guo, Da Zhao, Quan Zou, Fusheng Yu, Lijun Zhang, Lei Xu

**Affiliations:** ^1^ Institute of Fundamental and Frontier Sciences, University of Electronic Science and Technology of China, Chengdu, China; ^2^ School of Applied Chemistry and Biological Technology, Shenzhen Polytechnic, Shenzhen, China; ^3^ School of Electronic and Communication Engineering, Shenzhen Polytechnic, Shenzhen, China; ^4^ Beidahuang Industry Group General Hospital, Harbin, China

**Keywords:** *CDKN2A*, TMB, MSI, immune infiltrates, pan-cancer

## Abstract

The *CDKN2A* (cyclin dependent kinase inhibitor 2A/multiple tumor suppressor 1) gene, also known as the *P16* gene, encodes multiple tumor suppressor 1 (*MTS1*), which belongs to the INK4 family. In tumor tissue, *CDKN2A* has a high expression level compared with normal tissue and reflects prognosis in tumor patients. Our research targeted the analysis of *CDKN2A* expression in 33 tumors and clinical parameters, patient prognosis and tumor immunity roles. The *CDKN2A* expression level was significantly correlated with the tumor mutation burden (TMB) in 10 tumors, and the expression of *CDKN2A* was also correlated with MSI (microsatellite instability) in 10 tumors. *CDKN2A* expression was associated with infiltrating lymphocyte (TIL) levels in 22 pancancers, thus suggesting that *CDKN2A* expression is associated with tumor immunity. Enrichment analysis indicated that *CDKN2A* expression was involved in natural killer cell-mediated cytotoxicity pathways, antigen processing and presentation, olfactory transduction pathways, and regulation of the autophagy pathway in multiple cancers. *CDKN2A* was significantly associated with several immune cell infiltrates in pantumors. *CDKN2A* may serve as a promising prognostic biomarker and is associated with immune infiltrates across cancers.

## Introduction

In recent years, malignant tumor incidence has increased year by year, and finding effective treatment methods for malignant tumors has been a research focus worldwide (Cheng et al., 2018; Zhao et al., 2019). At present, the effect of tumor treatment is unsatisfactory, and tumorigenesis is associated with gene variations. Mutations in cell cycle-related genes usually lead to tumor formation, such as *TP53*, *CDKN2A*, *RB1* and *BRCA1*.

The *CDKN2A* gene, also known as the *P16* gene, encodes multiple tumor suppressor 1 (*MTS1*), which belongs to the INK4 family. The *CDKN2A* gene was identified by yeast two-hybrid protein correlation screening for proteins that interact with *CDK4* (cyclin-dependent kinase 4) (Serrano et al., 1993). The *CDKN2A* gene, located on chromosome 9p21 and 8.5 kb in length, contains 3 exons and encodes a protein composed of 148 amino acids (Serra and Chetty, 2018). The P16 protein can bind to CDK4 and CDK6 (cyclin-dependent kinase 6) and inhibit the formation of kinase activity complexes by cyclin D (CD) and CDK4 (Yang et al., 1996). The kinase activity complex can prevent RB protein phosphorylation. Cells are stopped in the G phase by blocking the phosphorylation of RB protein and regulating the cell cycle. Mutation of the *CDKN2A* gene will remove the inhibition of the CyclinD-CDK4 complex. Then, RB protein phosphorylation results in abnormal cell cycle progression, and cells gain unlimited proliferation ability (Romagosa et al., 2011).

*CDKN2A* tumor suppressors with mutations or gene loss are related to various tumors. Methylation of the *P16* gene may be an important mechanism in the development of ovarian cancer. Compared with patients without *P16* promoter methylation, ovarian cancer patients with *P16* promoter methylation have a significantly higher risk of disease progression (Todd et al., 2000; Cheng et al., 2016). However, promoter methylation of P16 genes cannot be used as a marker in early ovarian cancer diagnosis (Jiang et al., 2017). Melanoma is a cancer formed by the gradual accumulation of pathological mutations in normal melanocytes. Loss of the *CDKN2A* gene may participate in early invasion and metastasis of melanoma and suppress the initiation of invasion through inhibition of BRN2 in melanoma (Zeng et al., 2018). The most common gene mutations in human pancreatic cancer are *Kras* activation and *P16* inactivation. According to research on human pancreatic cancer, the proto-oncogene *Kras* can induce the expression of *P16,* and blocking the induction of *P16* by *Kras* can lead to tumor transformation and cancer cell metastasis (Chang et al., 2014). The *CDKN2A* methylation frequency is significantly higher in pancreatic cancer patients, which is related to patient survival (Tang et al., 2015). Such genetic events in the *CDKN2A* gene may play an important role in pancreatic ductal carcinoma.

A homozygous deletion mutation of *CDKN2A* (P16) was found in 31 patients with 127 EGFR mutations in lung cancer treated with EGFR-TKIs. For patients with *CDKN2A* (*P16*) deletion mutations, the median progression-free survival was 5.3 and 10.5 months, respectively. Therefore, EGFR mutation with *CDKN2A* (*P16*) deletion mutation is associated with the development of lung cancer (Jiang et al., 2016). In a study of patients with lymphoma, 25% of *CDKN2A* (*P16*) deletion mutations and 22% of *TP53* deletion mutations were detected in tumor tissues. Patients with deletion mutations in both *CDKN2A* and *TP53* had an average survival of 1.8 years, significantly lower than 4.3 and 5.1 years for patients with deletion mutations in only one of the genes. Both *CDKN2A* and *TP53* deletion mutations are closely associated with shortened overall survival (Delfau-Larue et al., 2015). Thus, *CDKN2A* deletion mutation is a poor prognostic indicator in mantle cell lymphoma. Studies on malignant glioma have shown that deletion mutations of *CDKN2A* can be seen in 40.3% of cases, of which homozygous deletion accounts for 74%, and homozygous deletion is more common in patients with primary malignant glioma (Cen et al., 2012; Hu et al., 2021a).

*CDKN2A* is a multiple tumor suppressor 1 (MTS1) which encoded a protein named P16. P16 can bind CDK4 and CDK6. P16 protein inhibits cyclin D (CD) and CDK4 to form a complex with kinase activity then regulates the cell cycle. Phase G and cell cycle are abnormal if the *CDKN2A* gene is mutated or deleted, and the cells obtain unlimited proliferation ability. Therefore, the study of *CDKN2A* gene is very important to further understand the impact of gene mutation on the development of cancer. Here, we sought to determine the *CDKN2A* gene deletion status in 33 kinds of tumors, and through analysis of the *CDKN2A* gene and patient survival, correlations between clinical tumor stage, tumor mutation load, microsatellite correlation, the relationships between tumor microenvironment, immune cell infiltration, gene expression and enrichment of GSEA, the relationship between the *CDKN2A* gene and tumor progression was assessed.

## Data and Methods

### Data Acquisition and Processing

Thirty-three tumor transcription datasets, somatic mutation, and survival data were obtained from the UCSC Xena (http://xena.ucsc.edu/) database. There were 11,057 samples (10,327 tumor samples and 730 normal samples) for transcription data. The BiomaRt (version 2.44.4) package of R (version 4.0.2) was used for gene ID conversion. The dplyr (version 1.0.5) package of R (version 4.0.2) was used for *CDKN2A* gene extraction from the transcription data. To analyze the differential expression of the *CDKN2A* gene, the ggpubr (version 0.4.0) (https://CRAN.R-project.org/package=ggpubr) package was used in 33 tumors.

### Relevant Analysis of *CDKN2A* Expression and Prognosis of Tumor Patients

Overall survival status information for tumor samples (10,327) was downloaded from theUCSC Xena (http://xena.ucsc.edu/) database for 33 tumors. DSS (disease-specific survival), DFI (disease-free interval) and PFI (progression-free interval) status and time information were downloaded from TCGA Pan-Cancer (PANCAN) in the UCSC Xena (http://xena.ucsc.edu/) database. The survival status and time information for the 33 tumors were extracted and used for prognostic analysis. According to the *CDKN2A* expression level, two groups with high and low expression levels were distinguished, and the prognostic value of *CDKN2A* for the two groups in each tumor was analyzed using the OS (overall survival), DSS, DFI and PFI data. The *CDKN2A* risk ratio forest plot was plotted. The limma (version 3.44.3) (Ritchie et al., 2015), survival (version 3.2-10), survminer (version 0.4.9) and forestplot (version 1.10.1) R packages were used to perform this analysis.

### Analysis of *CDKN2A* Expression and TMB, Tumor Stage and MSI

The tumor microenvironment contains two components, immune and stromal cells, which possess diagnostic and prognostic value in cancers (Hanahan and Weinberg, 2011; Fridman et al., 2012; Zhang et al., 2019; Hu et al., 2020; Islam et al., 2020; Hu et al., 2021b). Stage information was obtained from the UCSC Xena (http://xena.ucsc.edu/) database for all tumor samples, and we divided the stages into 4. With all of the tumors, approximately 8099 samples were used for tumor stage analysis. The R packages limma (version 3.44.3) and ggpubr (version 0.4.0) were used for the analysis of tumor stages associated with *CDKN2A* expression. A total of 10,114 samples of 33 tumors for mutation data were used for tumor mutation burden (TMB) analysis. The Spearman correlation test and fmsb package (https://CRAN.R-project.org/package=fmsb) of R were applied for analysis of the correlation between the *CDKN2A* gene and TMB. A total of 10,415 tumor samples were used to calculate the MSI score combined with the *CDKN2A* gene expression data for MSI analysis. The Spearman correlation test method and fmsb package were also applied to create a radar plot between the tumor and *CDKN2A* genes.

### Analysis of the Tumor Microenvironment and *CDKN2A* Expression Levels

The tumor microenvironment is mainly composed of stromal cells and immune cells, which play an important role in cancer prognosis. ESTIMATE is a tool for predicting tumor purity in tumor tissues (Yoshihara et al., 2013). Estimate and limma packages were used to estimate tumor purity by calculating the proportion of immune and stromal cells based on 11,057 samples from 33 tumors. The correlations of *CDKN2A* expression and the tumor microenvironment score were tested by the Spearman correlation test method. The R packages ggplot2 (version 3.3.3), ggpubr (version 0.4.0) and ggExtra (version 0.9) were applied to determine the correlation distribution.

### Correlation Analysis of Immune Cell Infiltration and *CDKN2A* Expression

The 22 immune cell types found in tumors include naive B cells, naive CD4 T cells, memory B cells and resting memory CD4 T cells and so on (Newman et al., 2015). To estimate the infiltration of these 22 immune cell types, we used CIBERSORT (https://cibersort.stanford.edu/) to calculate their percentages based on RNAseq data (Newman et al., 2019). The limma (version 3.44.3) package was used for expression data processing, and CIBERSORT was used to compute these immune cell infiltration scores. Correlations between *CDKN2A* expression and the 22 immune cell infiltration scores were tested by the Spearman correlation test method. The R packages ggpubr (version 0.4.0), ggplot2 (version 3.3.3) and ggExtra (version 0.9) were applied to determine the correlation distribution.

### PPI Networks and Correlation of *CDKN2A* With Marker Genes

The *CDKN2A* PPI network was constructed in the STRING database (https://www.string-db.org/) (Szklarczyk et al., 2019), which contains 31 coexpressed genes. The 31 coexpressed genes, 1 tumor-associated gene and 47 immune checkpoint genes from the literature were applied to calculate the correlation with *CDKN2A* in 33 tumors with the Spearman correlation test and limma (version 3.44.3) package. The correlation heatmap of CDKN2A and 79 genes was constructed by the reshape2 (version 1.4.4) and RColorBrewer (version 1.1-2) packages.

### Analysis of Gene Enrichment

GSEA (genome set enrichment analysis) is a method to determine whether a set of genes shows concordant differences between two biological states. Thirty-three tumor expression datasets were separated according to the *CDKN2A* expression level. The phenotype label was *CDKN2A* expression level. Based on the KEGG database (https://www.kegg.jp/) (Kanehisa and Goto, 2000) and GO database, genes were enriched into clusters. In addition, normalized enrichment scores were applied to classify the enriched pathways (Subramanian et al., 2005). FDR <0.05 was used for the enriched standard. The R packages limma (version 3.44.3), org.Hs.eg.db (version 3.11.4), enrichplot (version 1.8.1) and clusterProfiler (version 3.16.1) were applied for GSEA.

## Results

### The mRNA Expression Levels of *CDKN2A* in Different Types of Cancers

**FIGURE 1:**
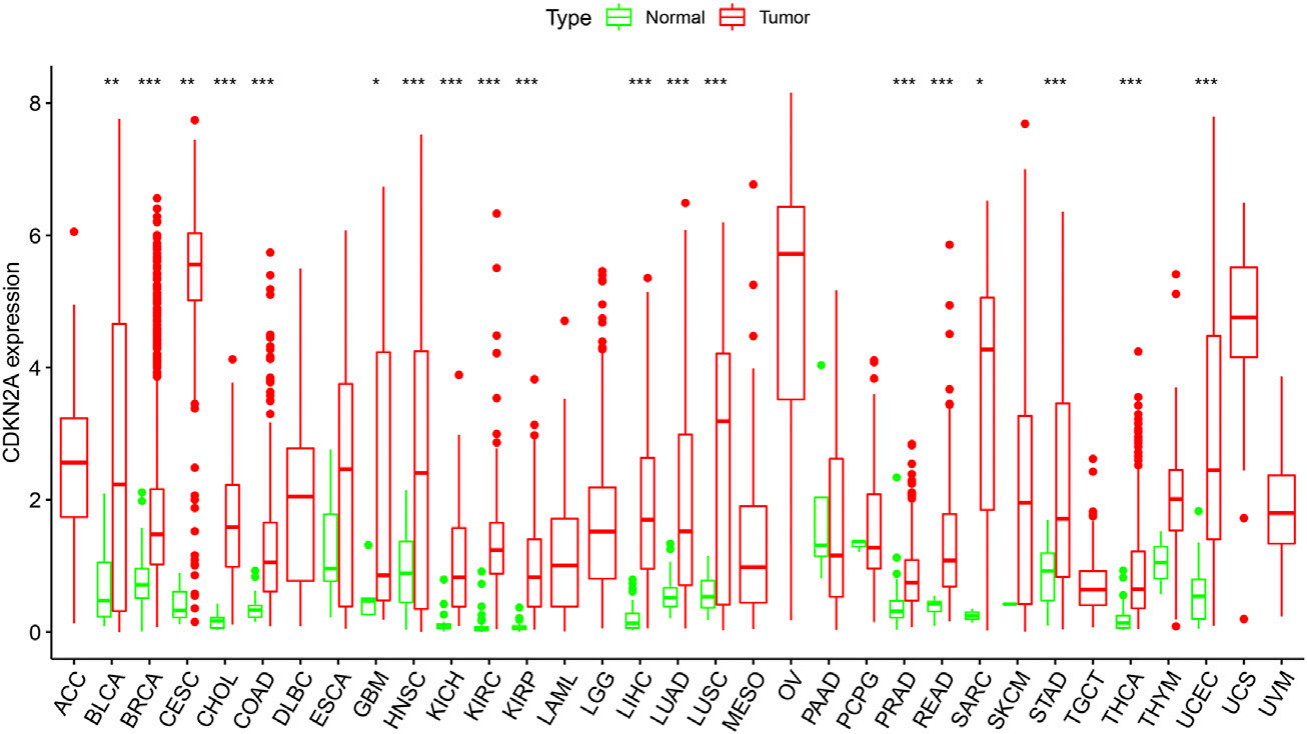
*CDKN2A* expression levels in different tumors. Expression of *CDKN2A* in tumors were performed by ggpubr (version 4.0.4) package of R (***p < 0.001, **p < 0.01, *p < 0.05).

To verify the differential expression of *CDKN2A* in tumor tissues, the *CDKN2A* transcriptome data of multiple cancers and normal tissues were analyzed using the ggpubr (version 4.0.4) package of R. This analysis revealed that the CDKN2A expression level was higher in various tumor tissues. Figure 1 shows the differential expression of *CDKN2A* in all 33 tumors and normal tissues. This analysis revealed that *CDKN2A* expression was extremely significant in 15 tumors compared with their normal tissue, including KICH (Kidney Chromophobe), BRCA (Breast invasive carcinoma), HNSC (Head and Neck squamous cell carcinoma), CHOL (Cholangiocarcinoma), COAD (Colon adenocarcinoma), KIRC (Kidney renal clear cell carcinoma), PRAD (Prostate adenocarcinoma), KIRP (Kidney renal papillary cell carcinoma), THCA (Thyroid carcinoma), LIHC (Liver hepatocellular carcinoma), STAD (Stomach adenocarcinoma), LUAD (Lung adenocarcinoma), UCEC (Uterine Corpus Endometrial Carcinoma) and READ (Rectum adenocarcinoma). In addition, SARC (Sarcoma), GBM (Glioblastoma multiforme), BLCA (Bladder Urothelial Carcinoma), LUSC (Lung squamous cell carcinoma) and CESC (Cervical squamous cell carcinoma and) tumors were significantly different from normal tissues. No change in expression levels was observed in the remaining 14 tumors. Abnormal expression of CDKN2A may be related to the occurrence of many cancers.

### Prognostic Value of *CDKN2A* in Pan-Cancer

**FIGURE 2:**
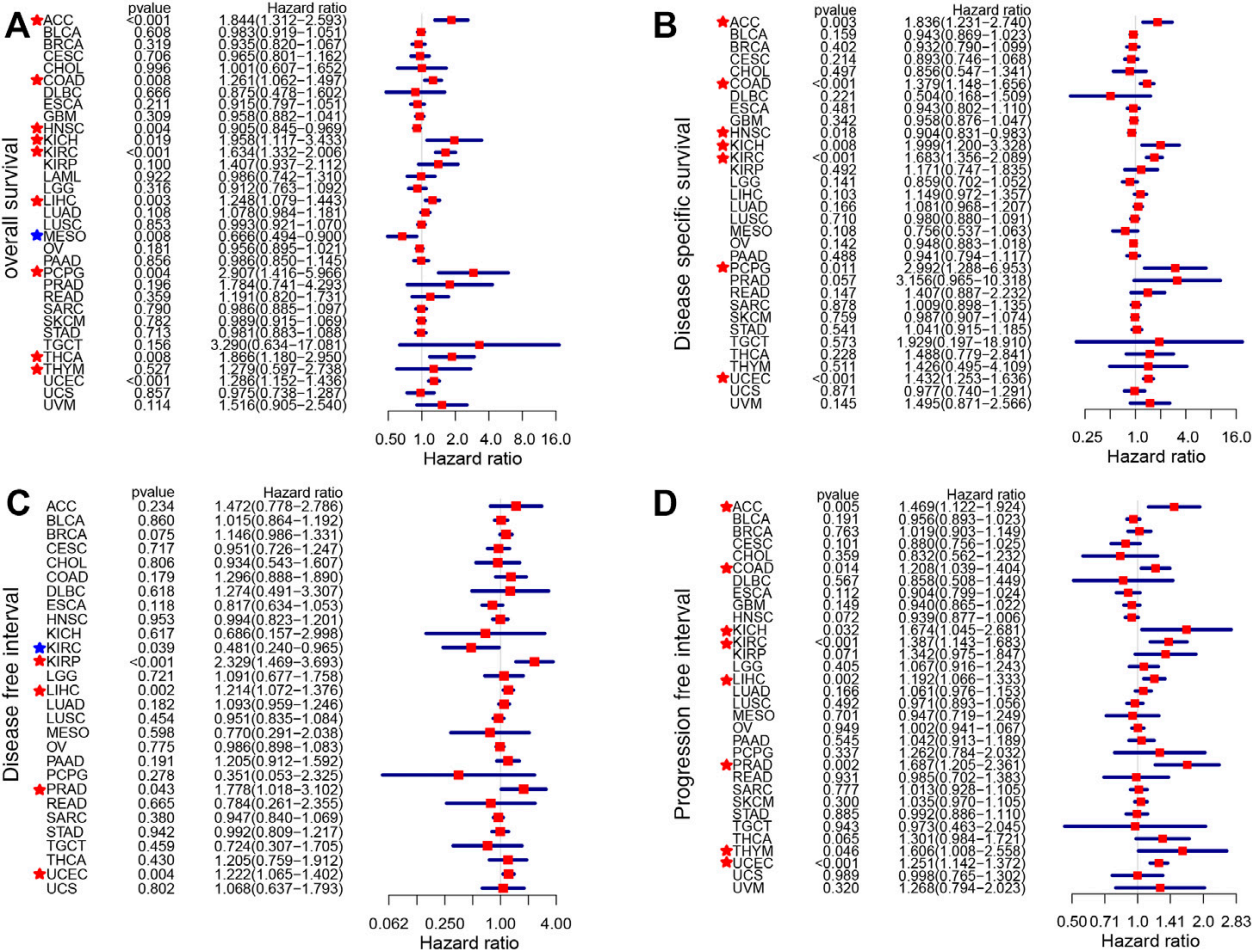
Survival analysis of *CDKN2A* expression in 33 types of cancers. **(A)** OS (overall survival) **(B)** DSS (disease-specific survival) **(C)** DFI (disease-free interval) **(D)** PFI (progression-free interval).

The impact of *CDKN2A* on the prognostic value of tumor samples was analyzed and compared with that of normal samples. *CDKN2A* with high expression was associated with OS (overall survival) in 9 tumors, and *CDKN2A* also correlated with poor DSS (disease-specific survival) in 7 tumors (Figures 2A,B). KIRP, LIHC, PRAD and UCEC tumors had poorer DFI (disease-free interval) due to the high expression level of *CDKN2A* (Figure 2C). Additionally, high *CDKN2A* expression was positively related to poor PFI (progression-free interval) in 8 tumors (Figure 2D).

**FIGURE 3:**
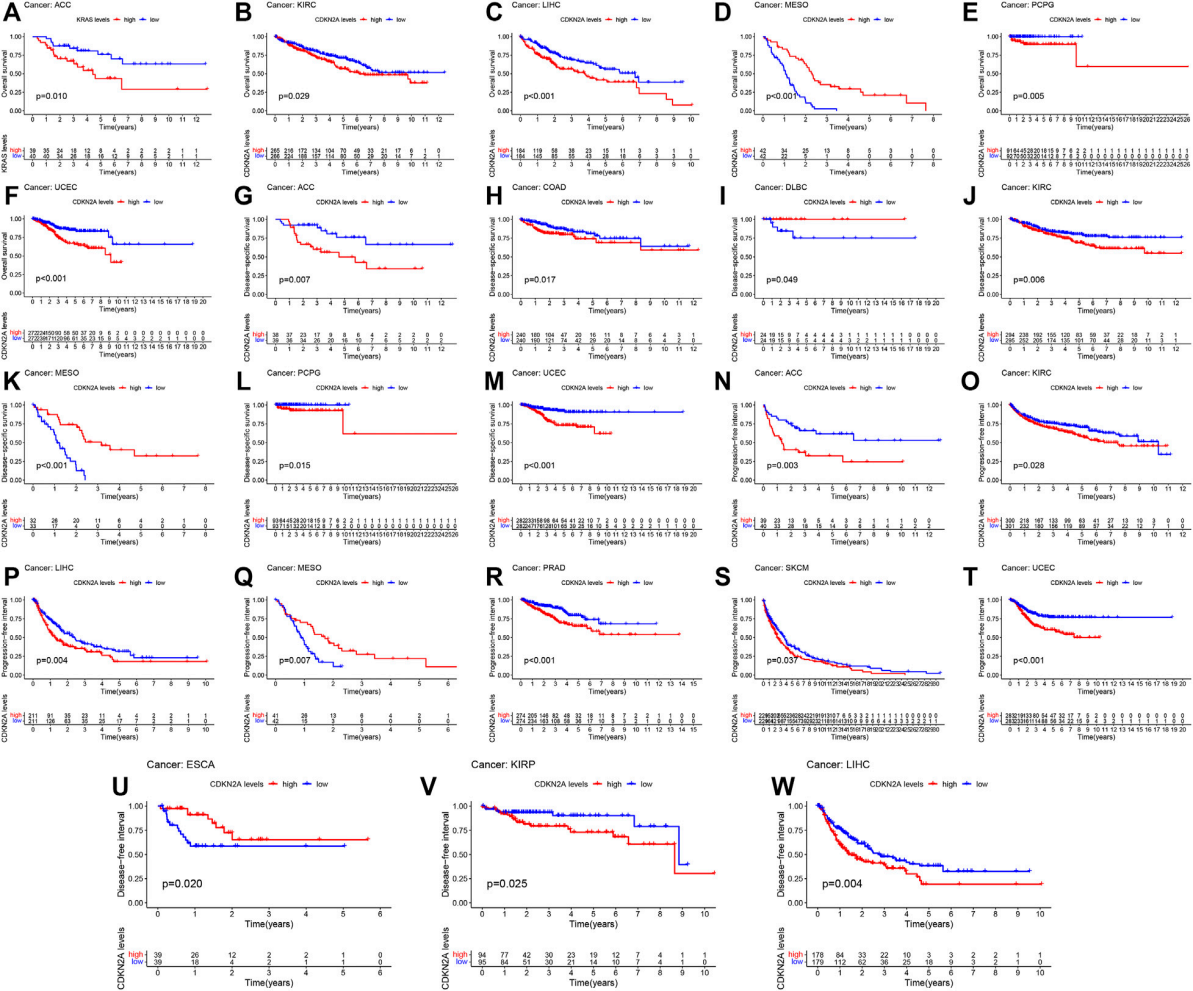
Relationship of *CDKN2A* expression and prognosis value in 33 cancers. **(A–F)** OS **(G–M)** DSS **(N–T)** PFI **(U–W)** DFI.

To further clarify the role of *CDKN2A* in a single cancer, clinical data for single cancers were used for analysis. Similarly, high mRNA levels of *CDKN2A* correlated with overall survival (OS) in ACC (Adrenocortical carcinoma), KIRC, LIHC, MESO (Mesothelioma), PCPG (Pheochromocytoma and Paraganglioma) and UCEC tumors, with *p*-values of 0.010, 0.029, 0.001, 0.001, 0.005 and 0.001, respectively. For disease specific survival (DSS), high mRNA levels of *CDKN2A* correlated with ACC, COAD, DLBC (Lymphoid Neoplasm Diffuse Large B), KIRC, MESO, PCPG and UCEC tumors, with *p*-values of 0.007, 0.017, 0.049, 0.006, 0.001, 0.015, and 0.001, respectively. The *p*-values of ACC, KIRC, LIHC, MESO, PARD, SKCM (Skin Cutaneous Melanoma) and UCEC tumors were 0.003, 0.028, 0.004, 0.007, 0.001, 0.037, and 0.001, respectively, due to the high expression level of *CDKN2A* in the progression-free interval (PFI). ESCA, KIRP and LIHC tumors were associated with high expression of *CDKN2A* for the disease-free interval (DFI), with *p*-values of 0.020, 0.025 and 0.004, respectively (Figure 3).

### *CDKN2A* Expression Levels Associated With the Clinicopathological Features of Patients With Tumors

**FIGURE 4:**
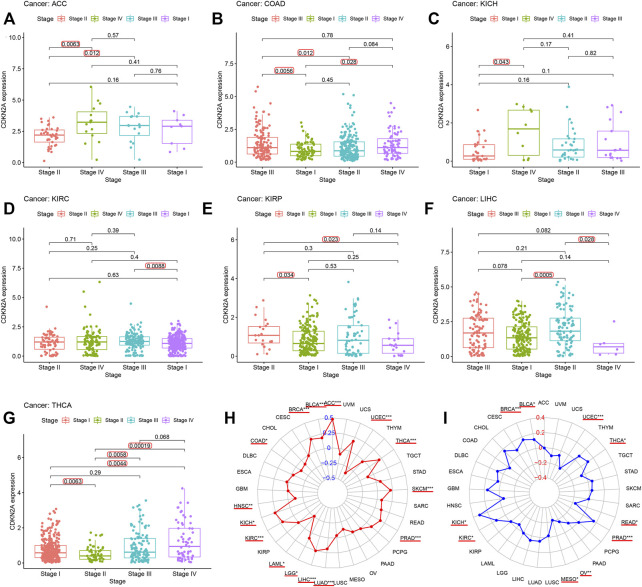
Relationship between *CDKN2A* expression level and clinicopathological characteristics. **(A–G)** Relationship of *CDKN2A* expression level and stage grade. **(H)** Tumor mutation burden (TMB). **(I)** Microsatellite instability (MSI). *, *p* < 0.05. **p < 0.01, ***p < 0.001.

TMB (tumor mutation burden) and MSI (microsatellite instability) status are related to *CDKN2A* expression for the clinicopathological characteristics of tumor stage pathology grade. To investigate this relationship, 33 tumor sample data points from the database were downloaded for analysis. Figures 4A–G shows that there was some effect on the high expression level of *CDKN2A* in seven tumors at several stages, specifically ACC, COAD, KICH, KIRC, KIRP, LIHC, and THCA. Moreover, *CDKN2A* with high expression levels in 10 tumors, BLCA, ACC, BRCA, THCA, PRAD, UCEC, LUAD, KICH, SKCM, KIRC, LIHC, and HNSC, was significantly correlated with TMB (Figure 4H), and high expression levels of *CDKN2A* in BRCA, UCEC and PRAD tumors were also significantly correlated with microsatellite instability (MSI) (Figure 4I).

### *CDKN2A* Expression Levels Associated With Tumor Microenvironment

**FIGURE 5:**
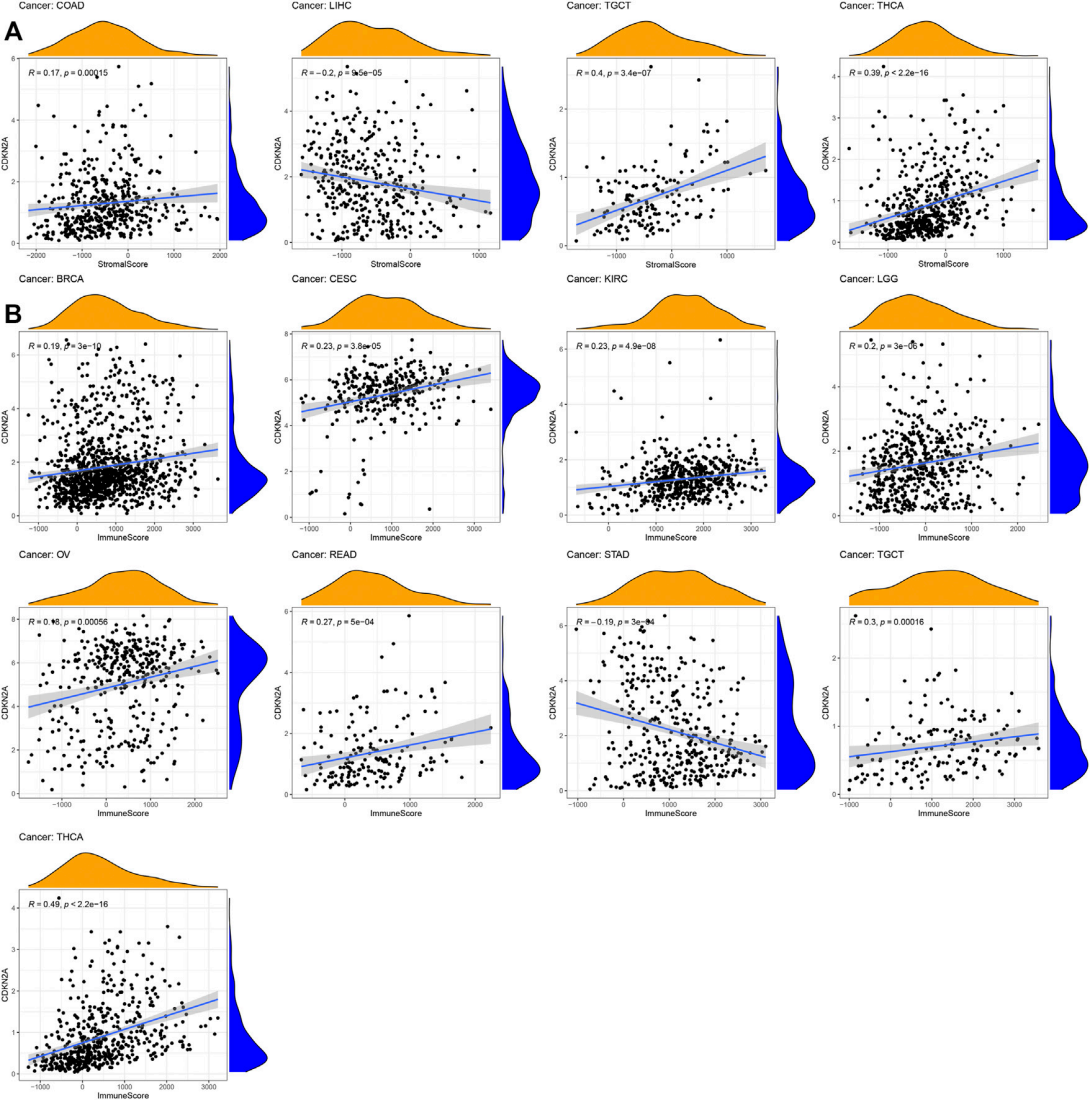
Relationships between *CDKN2A* expression and tumor microenvironment. **(A)** Stromal score, **(B)** Immune score.

For tumor microenvironment analysis, the estimate package of R was used to calculate the immune score and stromal score based on the *CDKN2A* expression level. Thirty-three tumor sample data points from the database were downloaded for analysis. As shown in Figure 5, the stromal score of LIHC and immune score of STAD were negatively correlated with *CDKN2A* expression levels. However, the immune scores of BRCA, CESC, KIRC, LGG (Brain Lower Grade Glioma), OV (Ovarian serous cystadenocarcinoma), READ, TGCT (Testicular Germ Cell Tumors), and THCA and the stromal scores of COAD, TGCT and THCA were negatively correlated with the *CDKN2A* expression level. The results showed that all immune and stromal scores of these tumors were significantly correlated with the *CDKN2A* expression level.

### The Association of *CDKN2A* With Tumor Immune Cell Infiltration Levels

**FIGURE 6:**
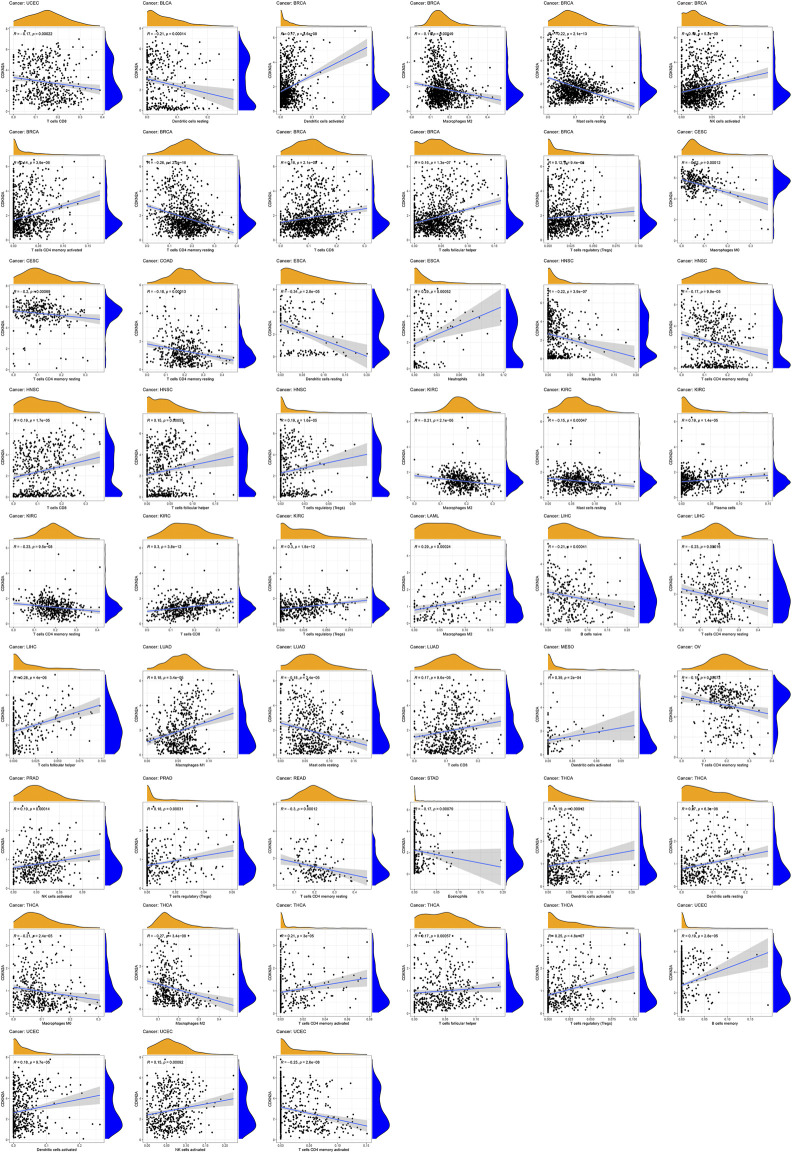
Relationships between *CDKN2A* expression and different types of immune cells infiltration level in tumors.

To investigate the relationship between immune cells and *CDKN2A* expression in tumors, 22 immune infiltrating cell expression datasets were prepared from the CIBERSORT website (https://cibersort.stanford.edu/runcibersort.php). High *CDKN2A* expression levels were positively associated with memory activated CD4 T cells, CD8 T cells, activated NK cells, regulatory T cells (Tregs), activated dendritic cells, and follicular helper T cells and were negatively related to the resting immune cell levels of mast cells, M2 macrophages, and memory resting CD4 T cells in BRCA. In HNSC, high *CDKN2A* expression was positively correlated with CD8 T cells and regulatory T cells (Tregs). High *CDKN2A* expression in KIRC was positively correlated with regulatory T cells (Tregs) and CD8 T cells and negatively correlated with M2 macrophages, resting memory CD4 T cells, and resting mast cells. *CDKN2A* expression in THCA tumors was positively associated with activated dendritic cells, regulatory T cells (Tregs), resting dendritic cells, activated memory CD4 memory T cells, and follicular helper T cells and negatively correlated with M0 macrophages and M2 macrophages. Furthermore, the results indicated that high expression of *CDKN2A* was negatively correlated with resting-state immune cells and positively correlated with the activation of immune cells in multiple tumors (Figure 6).

### Functional Analysis of *CDKN2A*

**FIGURE 7:**
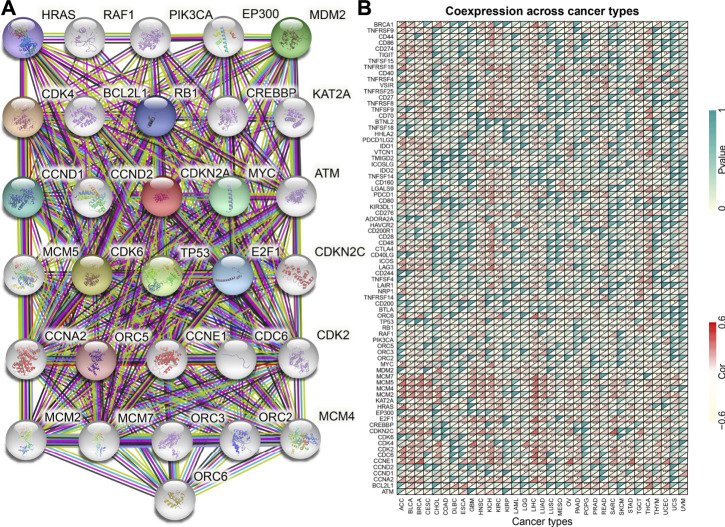
*CDKN2A* protein-protein network and expression relationships between *CDKN2A* and related genes. **(A)** PPI network for *CDKN2A*-interaction genes. **(B)** Correlation between *CDKN2A* expression and related genes (immune checkpoint genes and interacted related genes) expression.

**FIGURE 8:**
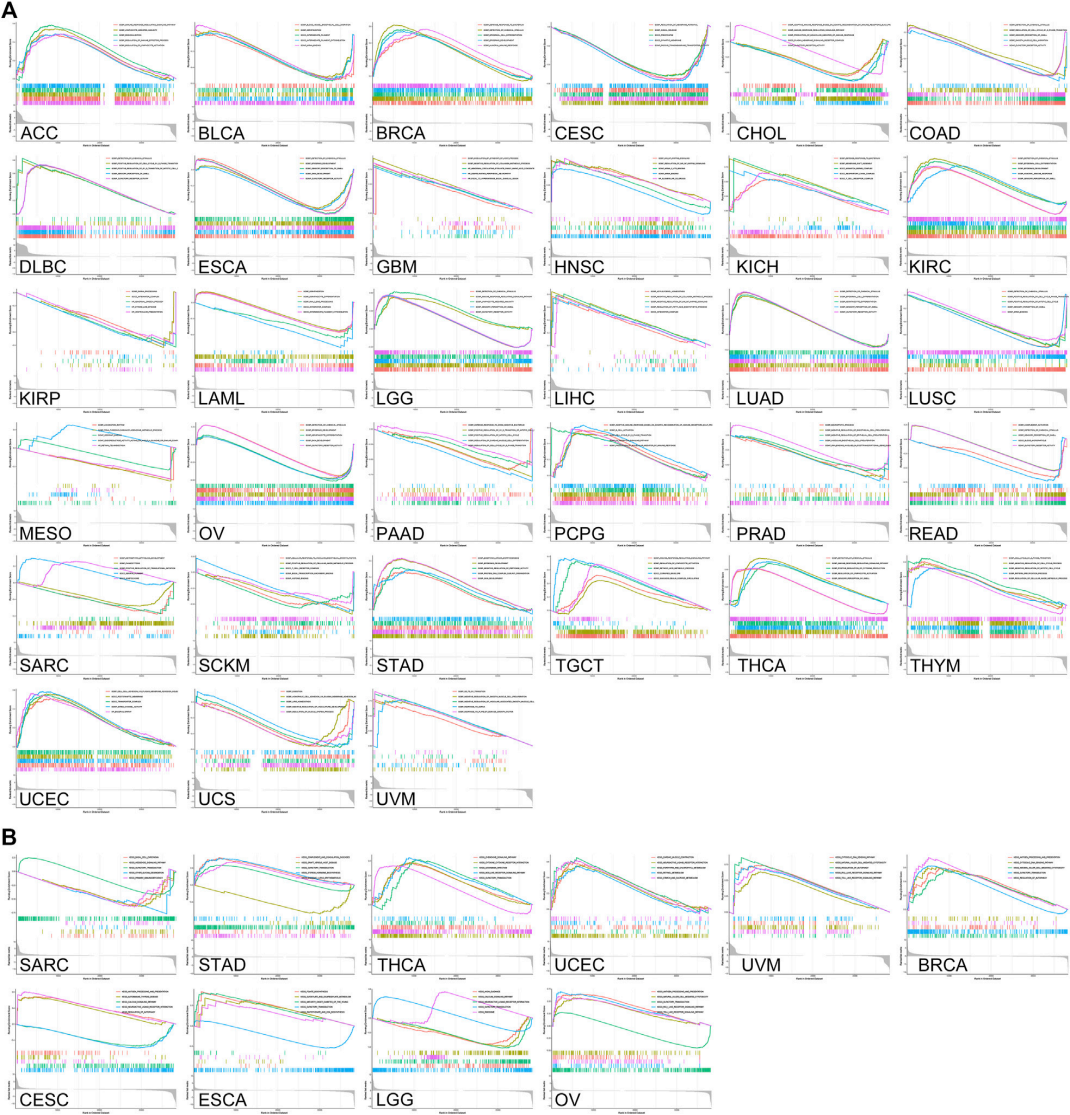
GSEA enrichment analysis of *CDKN2A* expression in 33 tumors. **(A)** GO enrichment analysis in various tumors. **(B)** KEGG enrichment analysis in various tumors.

The *CDKN2A* gene encodes multiple tumor suppressor 1, which regulates various cancers through different biological processes. The PPI network was used to investigate the molecular function of the CDKN2A protein using the STRING database (Figure 7A). The interacting genes from the protein-protein interaction network and common immune checkpoint genes were used to analyze the relationship with *CDKN2A*. The results indicated that several immune checkpoint genes and the genes from the PPI network were correlated with *CDKN2A* expression levels in many tumors (Figure 7B). GSEA was used for the further analysis of molecular mechanisms. High expression levels of *CDKN2A* are involves in the immune response, regulation of signaling pathways and regulation of immune effector processes in ACC. In BLCA, *CDKN2A* promoted blood vessel endothelial cell migration and mRNA binding. In addition, a high expression level of *CDKN2A* participated in epidermal cell differentiation, epidermal development and the humoral immune response in BRCA, KIRC and OV tumors. The T cell receptor complex is involved in KICH and SKCM tumors due to high *CDKN2A* expression. High expression levels of *CDKN2A* participated in keratinocyte differentiation and the intermediate filament cytoskeleton in LAML. High expression levels of *CDKN2A* participated in the immune response regulating signaling pathways and lymphocyte-mediated immunity intermediate filament cytoskeleton in LGG. In PCPGs, *CDKN2A* promoted B cell activation and the production of molecular mediators of the immune response. Circulating immunoglobulin complex and immune response regulating signaling pathways are involved in TGCT due to high *CDKN2A* expression (Figure 8A).

KEGG analysis suggested that a high *CDKN2A* expression level promoted many pathways in various tumors. In BRCA, CESE and OV, high *CDKN2A* expression levels are involved in antigen processing and presentation. High *CDKN2A* expression levels promoted natural killer cell-mediated cytotoxicity pathways in BRCA, OV and UVM (Uveal Melanoma). Olfactory transduction pathways are also involved in BRCA, OV, SARC, STAD, THCA, ESCA and LGG tumors. High *CDKN2A* expression levels promoted the regulation of the autophagy pathway in BRCA and CESC. In OV and UVM, high *CDKN2A* expression levels were involved in TOLL-like receptor signaling and the RIG-I-like receptor signaling pathway. *CDKN2A* has the potential to serve as a biomarker in various cancers (Figure 8B).

## Discussion

There are two proteins, p14ARF and p16INK4A, with tumor suppressive functions encoded by the *CDKN2A* locus (Sharpless and DePinho, 1999). The p16INK4A protein is a CDK (cyclin-dependent kinase) inhibitor, and it functions as a predominant suppressor of melanoma (Krimpenfort et al., 2001; Sviderskaya et al., 2002; Goldstein et al., 2006; Bennett, 2015). The expression level of the *CDKN2A* gene was higher in tumor tissue than in normal tissue. Furthermore, *CDKN2A* expression was significantly higher in 15 tumors, which was consistent with tumor stage analysis in COAD, KIRC, KIRP, KIHC, LIHC and THCA. Several studies have indicated the prognostic role of *CDKN2A* in many tumors (Christopher et al., 2002; Ai et al., 2003; Zeng et al., 2018; Christodoulou et al., 2020; Ji et al., 2020; Xande et al., 2020). Therefore, *CDKN2A* plays an important prognostic role in these tumors.

Recently, tumor mutation burden (TMB) has been highlighted as a positive predictive factor and immunotherapy biomarker for immune checkpoint inhibitors and participates in immune checkpoint blockade therapy (Chan et al., 2018; Lee et al., 2019). MSI-high status is usually used to predict tumor-agnostic markers and salvage immunotherapy in critically ill patients with end-stage cancer (Pietrantonio et al., 2020). The results indicated that TMB and high MSI were associated with high expression of *CDKN2A* in a variety of tumors, such as BLCA, BRCA, UCEC, THCA, PRAD, KIRC and KICH. The relationship of *CDKN2A* with MSI and TMB in this research is novel in these tumors, and *CDKN2A* is related to TMB and MSI and deserves more in-depth study.

Previous studies have shown that immune cells can recognize CDKN2A frameshift products (Hastings and Rausch, 2014). In mice, knockdown of *CDKN2A* can reduce IL-4-induced IL-10 production, and IL-4 can induce IL-10-producing CD8(+) T cells (Zhao et al., 2013). *CDKN2A* gene occurs more frequently in cell lines than in pancreatic cancer tissues. Such genetic events on the CDKN2A gene may play an important role possibly at a later step in the progression of pancreatic ductal carcinoma (Sugimoto et al., 1998). Zeng et al. find that complete CDKN2A loss coincides with the onset of invasiveness in melanocytic tumors at distinct progression stages (Zeng et al., 2018). p16 inactivation was the major mechanism of RB pathway perturbation in non-small-cell lung carcinoma, with homozygous deletion being the most frequent method, followed by methylation and the rarer point mutations (Tam et al., 2013). The detection of homozygous CDKN2A deletion by FISH would have been helpful in confirming a diagnosis of mesothelioma over reactive mesothelial cells in 12 of 13 samples with positive or suspicious cytology (Illei et al., 2003). For tumor immune cell infiltration, the results suggested that *CDKN2A* expression was positively related to CD8 T cells, NK activated cells and CD4 T activated cells but negatively related to memory resting CD4 T cells and resting CD4 T cells. High expression of *CDKN2A* can increase activated immune cell numbers and reduce resting immune cell numbers. Our research showed that *CDKN2A* was associated with the infiltration of multiple immune cells in BRCA, HNSC, KIRC and THCA. In these tumors, *CDKN2A* may be a potential immunotherapeutic biomarker to affect tumor cell viability.

The PPI network was analyzed in the STRING database, and 30 proteins interacted with CDKN2A, including CCND1, CDK1, CCND2, CDK2, CDK6, CCNE1, CDK4, TP53, MYC, CDC6, and HRAS. Most of the interacting proteins belong to cell cycle-associated proteins and proto-oncogene-associated proteins. The apoptotic pathway contains 10 core genes (*BAX*, *TP53*, *TP53INP1*, *CDKN2A*, *TP53BP1*, *CDKN1A*, *MDM2*, *CDKN1B*, *CCDN1* and *BCL2*), and these genes take part in the control of critical processes involved in hepatocellular carcinoma (HCC) (Yu et al., 2015). These genes may interact to control tumor progression. Disturbing the balance may result in a higher probability of tumor development (Cotter, 2009).

*CDKN2A* as a biomarker can be applied to assess prognosis in cancers. In addition, *CDKN2A* is expressed in various tumors and positively correlates with prognosis and immune cell infiltration, with high expression levels in most tumors. In general, the expression of *CDKN2A* played a detrimental role across cancers. In an analysis of immune cell infiltration, high *CDKN2A* expression was positively and significantly associated with many activated immune cells, which indicated that *CDKN2A* may be involved in tumor immunity. Together, these integrated analyses suggested that *CDKN2A* had prognostic value in cancers. Therefore, *CDKN2A* has potential as a therapeutic target for tumor treatment. However, our study promotes future immunotherapy research.

## Conclusion

Overall, we identified a novel cancer-related gene *CDKN2A*, which encodes *multiple tumor suppressor 1* (*MTS1*) and belongs to the INK4 family. *CDKN2A* expression levels, clinical parameters, patient prognosis and tumor immunity were investigated in 33 tumors. In addition, we also explored the association between *CDKN2A* expression level and tumor mutation burden (TMB) and microsatellite instability (MSI). However, *CDKN2A* expression is associated with infiltrating lymphocyte (TIL) levels, suggested that *CDKN2A* expression is related with tumor immunity. Based on these data, *CDKN2A* may be a promising prognostic biomarker with a potential molecular mechanism that affects survival outcomes in cancer patients.

## Data Availability

The datasets presented in this study can be found in online repositories. The names of the repository/repositories and accession number(s) can be found in the article/supplementary material.

## Glossary

CDKN2A/MTS1: cyclin dependent kinase inhibitor 2A/multiple tumor suppressor 1
TMB: tumor mutation burden
MSI: microsatellite instability
TIL: infiltrating lymphocyte
TP53: CDKN2A, RB1 and BRCA1.
CDK4: cyclin-dependent kinase 4
CDK6: cyclin-dependent kinase6
BRN2: POU class 3 homeobox 2
BAX: BCL2 associated X, apoptosis regulator
TP53INP1: tumor protein p53 inducible nuclear protein 1
TP53BP1: tumor protein p53 binding protein 1
CDKN1A: cyclin dependent kinase inhibitor 1A
MDM2: MDM2 proto-oncogene
CDKN1B: cyclin dependent kinase inhibitor 1B
CCDN1: cyclin D1
BCL2: BCL2 apoptosis regulator
GSEA: genome set enrichment analysis
UCSC Xena: Xena Functional Genomics Explorer
OS: overall survival
DSS: disease-specific survival
DFI: disease-free interval
PFI: progression-free interval
PPI network: protein-protein interact network
KEGG: Kyoto Encyclopedia of Genes and Genomes
GO: Gene Ontology
FDR: false discovery rate
ACC: Adrenocortical carcinoma
BLCA: Bladder Urothelial Carcinoma
BRCA: Breast invasive carcinoma
CESC: Cervical squamous cell carcinoma and endocervical adenocarcinoma
CHOL: Cholangiocarcinoma
COAD: Colon adenocarcinoma
CDKN2A: is a biomarker for multiple cancers
DLBC: Lymphoid Neoplasm Diffuse Large B cell Lymphoma
ESCA: Esophageal carcinoma
GBM: Glioblastoma multiforme
HNSC: Head and Neck squamous cell carcinoma
KICH: Kidney Chromophobe
KIRC: Kidney renal clear cell carcinoma
KIRP: Kidney renal papillary cell carcinoma
LAML: Acute Myeloid Leukemia
LGG: Brain Lower Grade Glioma
LIHC: Liver hepatocellular carcinoma
LUAD: Lung adenocarcinoma
LUSC: Lung squamous cell carcinoma
MESO: Mesothelioma
OV: Ovarian serous cystadenocarcinoma
PAAD: Pancreatic adenocarcinoma
PCPG: Pheochromocytoma and Paraganglioma
PRAD: Prostate adenocarcinoma
READ: Rectum adenocarcinoma
SARC: Sarcoma
SKCM: Skin Cutaneous Melanoma
STAD: Stomach adenocarcinoma
TGCT: Testicular Germ Cell Tumors
THCA: Thyroid carcinoma
THYM: Thymoma
UCEC: Uterine Corpus Endometrial Carcinoma
UCS: Uterine Carcinosarcoma
UVM: Uveal Melanoma
IL-4: interleukin 4
IL-10: interleukin 10
